# Possibilities and paradoxes in medicine: love of order, loveless order and the order of love

**DOI:** 10.1007/s11019-022-10093-0

**Published:** 2022-06-09

**Authors:** Thor Eirik Eriksen

**Affiliations:** 1 Department of Occupational and Environmental Medicine, University Hospital of North Norway, Tromsø, Norway; 2 Department of Community Medicine, Faculty of Health Sciences, UiT – The Arctic University of Norway, Tromsø, Norway

**Keywords:** Order, Order of order, Love, Explicate, Implicate, Symmetry, Asymmetry, Organic, Loveless, Psyche-soma, Disorder, The other

## Abstract

We have a desire to discover and create order, and our constitution, including our rational faculties, indicates that we are predisposed for such productivity. This affinity for order and the establishment of order is fundamental to humans and naturally also leaves its mark on the medical discipline. When this profession is made subject to criticism, frequently in terms of well-used reproofs such as reductionism, reification and de-humanisation, this systematising productivity is invariably involved in some way or other. It is, however, problematic that we rarely delve deeper and ask what order means, or reflect on its underlying, omnipresent and self-evident role. In order to approach this challenge, we initially and briefly place order in a conceptual and historical context. In what follows, we examine order explicitly, i.e. made an object of study, by taking a closer look at extensive multidisciplinary efforts to uncover the secrets of all its facets. Here we also try to identify some systems of order in medical science, including methodological and procedural order, which are indispensable as well as a source of problems. In the sections that follow, order is not defined as an explicit object of study, but comes to light in some exploratory and philosophising projects based on physics, mathematics and phenomenology . Each of these lets order and that which is ordered emerge in ways that may also shed light on opportunities and paradoxes in the medical domain. Key themes here include the Gordian knot of psyche – soma, the order of disorder and the patient as Other.

We adore chaos because we love to produce order.

- M.C. Escher.

It is indeed surprising that, despite the fact that the concept of

order is employed by philosophers in dealing with ontological,

epistemological, moral or political issues, so few philosophers

have bothered to focus their attention on the concept itself.

- A. Chmielewski

## Introduction

What is order? An easily recognisable and uncomplicated answer is given by Rotenstreich (1972), who writes, ‘Order is a state in which things are in their place or in their proper place’.^1^ The phrase ‘in their place’ can be linked to what the lexical sources refer to as ‘grades and ranks in an ordered or hierarchical structure’, ‘a rank, row or series’ and ‘the number of elements in a group’.^2^ That is, descriptions that in some way involve *regularity, rules, principles, standards or patterns*.^3^ When scientific studies follow a specific methodological design, tables show mutual relationships between meaning units or numerical values, symptoms are sorted and systematised into a diagnosis, conclusions logically follow from premises and arguments and physical laws lay the foundation for and explain nature; what they have in common is that they ensure in various ways that the things find or are assigned their rightful place. This means that their rightful place is in a *relation* – as components linked together in a certain pattern.^4^ What can also be seen to characterise these various expressions of order or the ordered is that in one way or another they concern humans’ determined and targeted process of meaning creation. This means what is organised for constituting and instituting order, what we can call for the time being ‘anthropogenic order’. This concerns how we create order *out of* the world, in order to live *in* it.

This creative aspect concern order that is produced and displayed by the human intellect, either through inventiveness and imagination alone or by utilising this capacity to identify order in nature or the universe. This fundamental concern with order, in all its manifestations, also has deep historical roots. Taking antiquity as a starting point, Aristotle states in his *Physics* that ‘ surely there is nothing *disorderly* in things that happen by nature or in accord with nature. For nature is a cause of order in everything’.^5^ The final sentence is crucial and normative in Aristotle’s work and pertains to an assertion that cannot be easily substantiated because it must be understood as a first principle.^6^ Many centuries later, through an interpretation of the Biblical narratives, Luther explains how we are incorporated in and subject to three systems of order or hierarchies: ‘the household [oeconomiam], the government [politiam], and the church [ecclesiam]’.^7^ The most critical of these is of course the last mentioned, which establishes how humans belong to an ecclesiastical order of creation in which they exercise compassion, patience and trust in God.^8^ Perhaps better known is Pascal’s (2011) distinction between the order of the body (or flesh), order of the mind (the intellect) and the order of the heart (order of charity).^9^ These are hierarchically ordered, with the body being the lowest and simplest level, while for Pascal the heart constitutes an infinitely superior order. In more modern times, we can also include Merleau-Ponty’s (1963) three forms of order (or structures): the physical (matter), the vital (life) and the human (mind). These orders all ‘represent different degrees of integration and, finally, must constitute a hierarchy in which individuality is progressively achieved’.^10^

This retrospective and very brief listing indicates that creating or establishing order has been a meaningful aim for humans. As Berrill (1966) suggests, we can therefore take our point of departure in the assumption that order is also an element of the human constitution: ‘Mankind searches for order in everything that he senses or contemplates. This search is a function of whatever mind is, for our minds see, impose and accept order’.^11^^,^^12^ This placement of order as a kind of a central hub for the human way of functioning and being is more recently confirmed by Chmielewski (2020), who in his discussion of the question of human nature he introduces the concept of *homo ordinans* – the ‘ordering-and-order-seeking being’.^13^ Taken as a whole, order can thus be thought to represent something we are “created for”, search for, are incessantly drawn to, work hard to produce and hold up as a central value or quality in our lives.

Let us now attempt to trace more concrete examples of ordering in human endeavour. This includes both the more directly charitable and productive aspects of our ordering activities and those order-related challenges and paradoxes that materialise as issues that in themselves appear to be anything but commonplace or ‘ordered’: that is, which can be seen as extra-*ordinary*, meaning something which is ‘out of the ordinary or regular course of order’.^14^ For example, we can see this unfold in organisational contexts where organisational charts, strategic plans and reporting systems, i.e. a form of *institutional order*, support the efficient production of services, but also have the potential to obstruct important aspects of human collaboration and ways of being.^15^ It appears even in the discipline of philosophy which assumes that knowledge production is carried forward by norms or ‘laws’ about the thinking process, such as the requirement for precision, clarity and logical rigour. 16 As Solomon (1999) suggests, however, this *order of thought* can lead to a philosophy that is ‘thin’, attenuated, emaciated, anorectic’.^17^ Finally, when we lift our gaze, we can see the contours of a far more dramatic scenario that concerns our relationship to and our dependence on nature. We are able to conduct responsible management in some areas, while in other ways we have become contributors to a climate crisis, in part because we have unwittingly put our trust in a *techno-industrial and unsustainable order*.^18^

In what will gradually follow, the main task will be to explore how the medical discipline encompasses some similar tensions and paradoxes. In such a project, however, the challenge may be that we quickly end up in a persistent assertion of the known conflicts between an *‘inhuman’ system-based medicine* and a *critical-humanist corrective* to the same. This corrective tends to be elaborated and reinforced by value-laden concepts such as mechanism, reductionism, objectivism and reification. We need, however, to continue to question and explore this allegedly problematic medical ‘systems thinking’ with a view to both adding nuance and understanding. A key contribution in this respect is provided by Svenaeus (2022). He highlights how this criticism, especially from phenomenology, ‘has mainly been identifying the back sides of objectification in medicine’.^19^ He therefore sets out to distinguish between ‘detrimental objectification and objectification that does not deprive the patient of his subjectivity’.^20^ Simply put, to distinguish between ‘good’ and ‘bad’ objectification. Like Svenaeus’ contribution, this paper is shaped by a desire to not only add nuance, but also to better understand the medical mindset. Here, however, we will not be concerned with objectification as such, but with an assumedly self-evident, inherent and non-thematised ‘underlying or extra quality’ that accompanies all objectifying endeavours, i.e. *order*.

The entry question is thus: What is order really like in its most basic actuality, and why is this question relevant to the medical discipline? Our exploration of such issues is structured as follows: In the *first section* we will treat the nature of order explicitly. This means that order, which is commonly included as a self-evident element of any exploratory project, itself is made into an object of inquiry. Here we will devote some space to an analytical approach in which differentiation and taxonomies are key aspects, and which revolves around some *a priori* norms and principles that are taken for granted, including those that assume that order (itself) can be subject to ordering principles.^21^With a view to the medical domain, a key task here will be to identify relevant formats of order and some appurtenant possibilities and challenges. An important reference point in this section is a contribution from the philosopher P. G. Kuntz. The subsequent three sections of the paper deviate slightly from this strategy to provide space for some philosophical ideas in which order is not defined as an object of study, but nevertheless remains relevant. Here, we challenge the idea that order not only comprises impartiality itself, the impersonal, non-partisan and universal nerve – i.e. that which is objective – but also that order (itself) is a thing that can be objectified. We thus remain open to ideas about order that do not necessarily appeal to humans’ industriousness or their productive and dominion-related objectives, but instead invite reflexivity, humility and our recipient capacities. The first of these explorations is based on the work of the physicist D. Bohm. His philosophical reflections have an ontological origin, touch upon the nature of the universe and things, and gradually lead us to a discussion of the Gordian knot of medicine: psyche vs. soma. The subsequent section includes contributions from the mathematician D. Orrell based on certain fundamental epistemological objectives in the sciences. His philosophical ideas revolve especially around the way in which such explicit objectives may conceal certain preferences of a mostly unarticulated and esthetical nature, gradually leading us to a discussion of the order/disorder dichotomy in medicine. The final section includes some phenomenological interpretations from the philosopher J. L. Marion and concerns how our understanding of objects and phenomena will necessarily also involve the topic of order. Here we will highlight a concern that in Marion’s view is decisive, and hopefully also for medicine, namely the order of charity.

## The orderliness of order

In the following, we shall pursue the question of the nature of order, or what we mean when conceptually speaking we use the category of order. As noted above, history abounds with examples of how this topic makes itself felt in some form or other. We could also mention Foucault’s (1966) contribution from the latter half of the previous century: *The order of things: An archaeology of the human sciences*, and the more recent addition from Pinter (2021): *Mind and the cosmic order. How the mind creates the futures & structure of all things, and why this insight transforms physics*. Although order is included as an integral part of a discussion linked to various historic eras (Foucault), or is highlighted in a discussion of the relationship between the mind and the physical world (Pinter), it is more rare to see order explicitly and separately placed on the agenda, as it is in the work of the philosopher G. Kuntz. In the 1960s he invited a number of researchers from a variety of disciplines to help explore this topic, which concluded with the book project ‘*The Concept of Order*’ (Kuntz, ed. 1968).^22^ The project was explicitly multi-disciplinary, but for Kuntz, the idea was not only to investigate notions of order across disciplines, but also to attempt ‘to engage in a search for a common element in the diverse uses of order’, with the caveat: ‘if there is one’.^23^

The book sets out to undertake a comprehensive analysis and review of the numerous aspects of order. According to Kuntz, the advantage of the analysis lies in the fact that it is ‘strong in discrimination of differences’ and helps us ‘to perceive the *manyness* of orders’.^24^ This strategy leads to a comprehensive presentation of various manifestations of order, including a) *historical forms of order*, which include ‘good order’ (eutaxia), order as arranged (taxis), mechanical order (Galilei, Descartes), rational order (geometry), Darwinian order (evolution), as well as b) o*rders of modernity*, which brings to mind the emergence of modern society and the identification of personal (agency of men), social, political and institutional order. Furthermore, Kuntz claims that we might well seek answers also in c) *disciplinary meanings of order*, including 1) religion, which reminds us of the presence of a divine order, but also refers to ‘the right ordering of human thought and action’, here understood as a teleological concept,^25^ and 2) *ethics*, which refers to a moral context in which order concerns ‘the right relations between persons’.^26^ The focus here is on ‘a normative order, that is, there are standards by which we judge the rightness and wrongness of acts’,^27^ 3) *aesthetics*, referring to an order which ‘regularly deals with arrangements of sensuous qualities’,^28^ and 4) *science*, which encompasses the most obvious formats of order, and where laws of nature and mathematics are the ruling principles. Here, order is closely associated with such key concepts as causality, determinism, mechanisms and explanations.

However, after this comprehensive review of the numerous facets of order, Kuntz finds that he must acknowledge that the sum of the contributions has been unable to identify a *common principle* for order or *a single world order*. Order is more rightly *orders*, in the plural, and the position taken by the majority of the contributors to the book is a kind of *pluralism of orders*.^29^ Kuntz further acknowledges that the analysis has its limitations and that ‘ordering order’ has proven to be a challenging project. Thus, the differentiation does not necessarily bring us closer to the ‘matter’ of order, and difficult metaphysical issues emerge at the fringes of the analysis. These include a confrontation with the opposite of order, i.e. chaos (the undifferentiated) and disorder (uncoordinated), the indivisible order of the divine, a possible meta-order, chance, freedom, spontaneity and creativity. Kuhn (1968), one of Kuntz’ co-authors, also highlights how challenging questions such as ‘how is man related to the order of the whole (the world)?’, and ‘how is man related to the order of the historical world?’ mean that we are again faced ‘with our initial dilemma, the breakdown of thought in matters of order’.^30^ What is indicated here is that every aspect of order that we succeed in ordering tends to be accompanied by a troublesome opposite or a newly emerging question that disrupts and upsets the same.

Given this brief outline of Kuntz’s contribution, we can here *keep order in our line of sight* and thereby get on the track of both a diversity of order formats and their accompanying challenges in the field of medicine. We know, for example, that the research branch of the discipline relies on a well-developed *methodological order*, sustained by qualities such as systematism, transparency, verifiability, objectivity, validity and reliability. In this context, the gold standard of the randomised, controlled trial (RCT) guarantees high-quality data and prepares the ground for so-called evidence-based medicine (EBM), but in this process it also produces a controversial ‘average randomised patient’ whom the clinical practitioner may only rarely encounter in his practice.^31^ The diversity of studies that are included in this search for reliable data can be further ranked in an evidence hierarchy, an arrangement that we can describe as an *order of evidence*. This differentiation distinguishes between strong and weak studies and constitutes an important guideline for medical practitioners, but is also criticised for leading to dogmatism, scientism and a pseudo-religious belief in science that is itself not scientific.^32^ Backed up by this source material, the clinician can rely on various tests and measurements that follow standard procedures and are able to detect anomalies. These are part of what we can describe as a formalised *procedural order* that in many cases provides vitally important answers, but in its habitual application also may lead to burdensome and costly overtesting and unnecessary medicalisation of the population. Moreover, the clinician is dependent on identifying and diagnosing specific diseases. Indispensable tools for this are manuals such as the ICD-10 and DSM-5, which guarantee an all-important clarity about disease in what constitutes a required necessary *diagnostic order*, but also serve as a reference point that can distract us from significant and non-objectifiable life expressions in a suffering person.^33^ Furthermore, the clinician can consult medical guidelines that give access to a quality-assured *authoritative and consensual order*. The risk, however, is that even such guidelines can be reduced to recipe books, whereupon we fail to acknowledge that they may have limitations in their availability and applicability in the local context.^34^ In the final account, and in the communication and contact with the patient, the doctor has access to a number of written techniques and tools that are included in what we here can refer to as a *communicative order*.^35^ These tools can be essential reference points in clinical encounters, but alone they will not guarantee that the suffering patient feels welcomed, encompassed, respected or understood.

The described areas that represent opportunities and problems for the field of medicine refer to some well-known discourses that have emerged in recent decades. Somewhat simplified, on the one hand we find some systems of order that guarantee safety, security, predictability and quality in the health services, which obviously help promote some of the fundamental trust that we place in medicine. On the other hand we can see the outline of how exactly this affinity for systems, technologies, manuals and instruments, or what we here can refer to as a *love of order*, can become so dominant that it comes at the cost of the human aspects, care, presence and ethics, or what we may describe with a concept from Pascal as the *order of charity* or the *order of love*. These problematic aspects remind us that the orders and the formats of order that are institutionalised in medicine may also have their limits. In this context, it is particularly meaningful when Lorand (2000) reminds us how ‘everyday experience teaches that the world does not equally accept every kind of order that we seek to impose on it. Sometimes the world resists, and there are various degrees of resistance that may teach us something about the world that *exists beyond our will and desired orders'*.^36^ In other words, our order-making projects can be taken too far, and our inclination towards system and structure have the potential to camouflage certain aspects of our lifeworld and reality.

So what are we left with, given these introductory descriptions and clarifications? First and foremost, we gain access to a multi-faceted overview of different dimensions, categories and formats of order. This means that we have used certain immanently structuring principles of order, i.e. the assumed meaning of this concept, on the matter, thing or object of order itself. The challenge associated with such a strategy is that we ‘*see order’*, because order is what we quite naturally are ‘*looking for*’. In other words, the *gaze* that sweeps over the world and that departs from order as a universally organising principle finds imprints of it no matter in what direction it glances. In this context, although used figuratively, the German saying ‘Alles in Ordnung’ (everything is in order) may have particular resonance. The approach used by Kuntz and his colleagues can be regarded as methodologically adequate, although it pays insufficient attention to the way in which order, i.e. the *object of study*, in itself and of itself represents an active component that already leaves its imprint on the exploratory effort before it is presented in its innumerable manifestations. This concerns how order shapes thinking (since it is the foremost resource of reason (ratio)), as it ensures the logical sequence of the description and is a principle that enables the structuring and classification of orders of modernity, disciplinary order, religious, ethical, aesthetic and scientific order in the first place. Summing up, order is not only what we are looking for and what we are seeing, but also what we are seeing *with* or *through*. Following Rotenstreich (1972), we can somewhat more pointedly state that this concerns how order can have the role of both ‘an *a priori* assumption of science’ or more crucially ‘*the horizon of the intentionality of science’*.^37^ The problematic aspect of this is thus not a matter of the human’s ability to *produce order*, but rather the absence of reflection about the issue that we may ourselves be deeply *embedded in*, or even *surrendered to*. In the following, we shall explore some aspects of this ‘embeddedness’ more closely.

## Rethinking fragmentation—introducing explicate and implicate order

In our daily lives, whether in the private or professional sphere, we are highly dependent on structuring, abstraction, categorisation and systematisation in one form or another. These divisive practices are a feature that also characterises what we can understand as the order-producing tools of the sciences: *theories, models and methods*. They enable stabilisation and fixation, produce a necessary overview and enable ‘ordered’ action and intervention. They have proven to be successful; that is, they have accompanied the creation of ground-breaking scientific results that represent significant advancements for humanity, especially in the branch of medicine. We are therefore dependent on such tools and this systematic way of working, while they can delude us at the same time. One of these delusions concerns how we may think that the framework, i.e. the models, *are* reality itself. We can forget that through the models we only *see* those aspects of reality that the models’ beam of light allows us to see.^38^ We may also *act on*, control and manipulate a section of the world (nature) on the basis of model-based knowledge without taking the range of potential consequences into account-consequences that only become visible if we can have a perspective on the *‘whole’*, no matter how problematic and controversial such an idea is.

Within the natural science tradition, it is probably the works of the David Bohm in particular that most directly address such issues and radically challenge established ideas about order.^39^ Based on his background in theoretical physics, quantum physics and quantum mechanics, he has developed over time what we can understand as a radical *ontological* or *metaphysical* re(interpretation) of quantum theory.^40^ An important starting point for Bohm is that ‘we are largely unaware of the degree to which *inherited orders*, or paradigms, dominate our perception and thought’.^41^ What in Bohm’s eyes is the far-reaching problem, what one faces across disciplines, has to do with how these orders require *fragmentation*. Bohm briefly expands on this idea in an interview from 1990: ‘I think the difficulty is this fragmentation, first of all. All thought is broken up into bits’.^42^ That is, ways of thinking that do not take into account that ‘we are internally related to everything’.^43^ In his book *Wholeness and the Implicate Order* (1980), he elaborates on these thoughts: ‘Being guided by a fragmentary self-world view, man then acts in such a way as to try to break himself and the world up, so that all seems to correspond to his way of thinking’.^44^ This means that we ‘see’ a world consisting of objects that are external in relation to each other. What we do not understand is that by taking this approach, we have *changed the very phenomenon being studied*. The end-product of our analyses, portrayed as lawfulnesses and based on mathematical formulas, does not take into account ‘the nature of reality in general and of consciousness in particular as a coherent whole, which is never static or complete, but which is in an unending process of movement and unfoldment’.^45^ This coherent whole concerns a deeper reality, an unbroken wholeness. A wholeness from which a new order arises: *the implicate or enfolded order*. Bohm writes the following about this:

In the enfolded order, space and time are no longer the dominant factors determining the relationships of dependence or independence of different elements. Rather, an entirely different sort of *basic connection* of elements is possible, from which our ordinary notions of space and time, along with those of separately existent material particles, are abstracted as forms derived from the deeper order. These ordinary notions in fact appear in what is called the *explicate or unfolded order*, which is a special and distinguished form contained within the general totality of all the implicate orders.^46^

Here Bohm emphasises that the explicate or unfolded order, the abstracted order, is a derivative of the fundamental and overarching *enfolded order* (the implicate order).^47^ The former assumes that all things can be reduced to *explanations* in the same order format (based on prevailing scientific standards and principles in the various natural science disciplines). The implicate order is equally *immanent* in these explicate expressions of order. The main reason we do not pay attention to the deeper underlying order is, according to Bohm, that we ‘have become so habituated to the explicate order, and have emphasised it so much in our thought and language, that we tend strongly to feel that our primary experience is of that which is explicate and manifest’.^48^ Especially challenging for our established way of thinking is that Bohm ascribes the implicate order to both *matter* (living and non-living) and *consciousness* (mind). They are inextricably bound; they rest on common ground.

These basic ontological assumptions have far-reaching implications. A striking and obvious example is the indelible paradox referred to in the medical field as *psyche-soma* (also referred to as the mind-body split). This refers toa division or fragmentation that still governs both the organisation of the health service and medical research. Wording such as ‘comorbid psychiatric disorders *and* medical disorders’, ‘physical *and* psychological factors’, or psychological mechanisms and physiologic functions, certainly clarifies that there is a *relationship*.^49^^,^^50^ For Bohm, however, this is inadequate, and he emphasises that ‘such a meaning is not compatible with the implicate order. In the implicate order we have to say that mind *enfolds matter* in general and therefore the body in particular. Similarly, the body enfolds not only the mind but also in some sense the entire material universe. (…both through the senses and through the fact that the constituent atoms of the body are actually structures that are enfolded in principle throughout all space.)’^51^ Most importantly in this context is that what we choose to call ‘psyche’ and ‘soma’ only concerns different aspects of *one whole and unbroken movement*. That is, an *unbroken whole*, ‘a mutual enfoldment’ outside of our field of view when we refer to how the ‘psychological symptoms’ interact with the ‘somatic symptoms’.^52^ A central nexus in Bohm’s work thereby relates to the difficult and highly controversial concept of holism.

With varying degrees of success, medicine has attempted to untie this Gordian knot and develop the profession in a somewhat more holistic direction. We can find traces of this in titles such as ‘*Time to move beyond the mind-body split’* (Bracken 2002); ‘*Beyond the Mind-Body Dualism’* (Zacharacopoulou, 2006); ‘*Mind-body dualism: a critique from a health perspective′* (Mehta (2011) and ‘*About the psyche-soma unit: The “knowledge” of the body. Projective and clinical implications’* (Sola, 2020). The challenging task these authors set out to address is to unite what has been inconveniently separated. Perhaps the most groundbreaking innovation in the field of medicine in this respect is referred to as *psycho-neuro-endocrino-immunology (PNEI)*. The hyphens signify that an attempt is made to merge these sub-disciplines that have previously operated within separate spheres, i.e. psychology, neurology, endocrinology and immunology. The assertion, as suggested by Bohm, that all this should refer to one whole and unbroken movement, no longer appears to be a radical and distant holistic utopia. The most recent contribution in this area, that of the Italian doctor and PNEI researcher Lissoni et al. (2021) suggests exactly this. The book’s title, *The Clinical Psychoneuroendocrinoimmunology: PNEI, The Real Holistic Medicine*, is in this respect indicative. The authors emphasize, as expected “the impossibility to separately consider the functionless of the three major regulatory systems., since they are connected by reciprocal influences.” 53^,^^54^

Although PNEI represents the solid, scientific and experimental basis for a more holistic mindset in medicine, we can also find traces of this in the development of models inspired by the humanities in this discipline, i.e. contributions that pull in the direction of what is here interpreted as Bohm’s ontological or metaphysical holism. These manifest themselves in initiatives that ask the medical discipline to acknowledge the *patient’s lived experience*, as a *person* (patient- or person-centred medicine), the patient’s *narratives* (NM – narrative medicine) and the *Bio-Psycho-Social* connection that the person is a part of (e.g. the BPS model).^55^ A general feature of such initiatives is that they originate from a critique of an unnecessary or exaggerated *fragmentation* of medicine. What also characterises such works is that they seek an *underlying or fundamental whole or totality*, in the patient or in their environment, that cannot necessarily explain (or give a causal explanation of) the patient’s symptoms or condition, that is, a whole outside of the medical ‘extracts and crystallisations’ that diagnoses represent or the explicate order in which the biological laws are essential constituents. To the extent that we accept Woods’ (2015) simplified description of holism as “A theory that parts of a whole are so interconnected that they cannot exist independently of the whole or cannot be understood without reference to the whole”, we see the contours of how Bohm’s work and the more recent initiatives are somehow related, despite the profound differences in approach and scholarly foundation.^56^

In conclusion, it can be mentioned that these proposals from Bohm have garnered criticism. One objection concerns the demand for scientific or epistemic order and notes that Bohm does not present sufficient (experimental) evidence for the existence of an implicate order.^57^ Another, related criticism is that he uses a foreign, or even esoteric, language that is not grounded in physics and quantum physics. We cannot ignore such objections, but many of them elicit scholarly discussions among qualified voices within quantum physics. In this paper, however, the focus has been on Bohm’s desire to *philosophise*, which led to the development of ideas that extend beyond the disciplines. We have been interested in his *general philosophy*, which according to Seager (2018) ‘goes far beyond the familiar but perennially peculiar non-locality and entanglement of quantum systems’.^58^ It is precisely this capacity to philosophise that has made an impression in modern times, including through the recognition of Bohm as a pioneer of the philosophical initiatives known as panpsychism. Accordingly, his work is included in radical ontology, or what we can describe as ‘extremely radical metaphysics of nature’.^59^ In light of this, we can understand the articulation of implicate order as something other than ‘yet another order we have put in order’ or an ‘order that we are awaiting proof of’. This represents instead a potential source of disruption of our entire worldview, which *can* give meaning both in our encounters with other people (as in medicine) and as we are now facing an ongoing climate crisis.

## Rediscovering the beauty of order

The attraction towards order, that which has structure and that which helps to ensure that ‘all things find their proper place’, is difficult to disregard as a characteristic of the human way of thinking and being. It is an attraction that usually, and not seldom of necessity, is bound by rational, instrumental, objectifying and results-oriented preferences. The question, however, is whether we, in our judgment and criticism of this fragmentary way of thinking which attests to positive certainty and control, at the same time overlook the possibility that people are attracted to order for other reasons as well. Such questions have been the subject of closer scrutiny by the mathematician Orrell (2012) in his book *Truth or Beauty: Science and the Quest for Order*.^60^ With regard to the possible aims of scientific investigation, he clarifies rather early on that both the desire for control and positive certainty (truth) must necessarily be acknowledged. However, what lies in the background is an historically based and inescapable, motivating scientific aim: *beauty*. 61 More specifically, this entails an ideal of beauty focusing on the *straight, harmonious, symmetrical and static/stable, * that is a kind of machine aesthetic in which *elegance* is crucial point of reference. 62 These aesthetic effects have had a calming effect through the ages, i.e. they are part of a *calming kind of order*. Here Orrell refers to Democrit’s theory of atoms, and emphasises that ‘Like a painting of a beautiful sunset, or a pleasant wallpaper, it gave a reassuring sense that everything *was in its place*’.^63^ Thus, scientific theories are only seemingly in the driver’s seat. Orrell explains:

Even deeper than theories, though, is aesthetics. The way that we see the world has been shaped by the traditional scientific aesthetic. We look for reductionist theories that can break a system down into its components. We seek elegant equations that can be rigorously proven using mathematics. We aim for a unified, consistent theory. We celebrate symmetry, clarity, and formal beauty. Predictive accuracy is supposed to be the test of reductionist science - but when accurate predictions prove impossible, aesthetics wins out every time. What we mean by ‘good science’ is strongly related to what we (i.e., scientists) mean by beauty.^64^

What Orrell is suggesting here deals with how a fundamental motivation for theory development, mathematical application and reduction rests in aesthetic preferences that are seldom formulated explicitly. The question, however, is to what extent preferences for certain aesthetic qualities in the sciences, expressed or unexpressed, are a problem. At a more general level, Orrell answers this question on the basis of two concepts: *the standard model* (physics) and *the perfect model*. The former deals with theoretical physics and has its origins in the 1960s and 1970s. The never-ending search for increasingly fine-tuned details and improvements on the model’s parameters, *represses* more urgent questions that deal with ‘how we can learn from the fine-tuning that we see in natural systems… and apply that knowledge to our own behaviour. The harmony we need is not with the spheres or the strings, but with the *world*—and at the moment, we are badly off-pitch’. According to Orrell, this concerns our thoughtless exploitation and manipulation of nature under the influence of a machine aesthetic that devalues nature as a ‘a living wonder’.^65^ ‘The perfect model’ concerns not only theoretical physics but also an ideal referred to within a number of different disciplines. The advantage of this model is that within various disciplines, whether it is meteorology, health, medicine or economics, it appears as a ‘flawless representation of reality’.^66^ He even goes so far as to claim that large segments of the scientific community suffer from the *perfect model syndrome*.^67^ One is continually in search of confirmation of the model’s excellence through increasingly refined explanations. According to Orrell, the crucial problem we are once again confronted with is how ‘the models have become confused with reality′.^68^ Although these are constituted and virtual copies of the world, we nonetheless manage to be spellbound. ‘The more perfect it appears, the greater the illusion’.^69^ On a bad day these models do not help to expand our horizon of understanding, but instead become an obstruction. Of course, this critique does not disregard the explanatory power or the aesthetic qualities of a model design, but only suggests that the idea of the model’s perfect depiction of reality *can also* lead us astray.

Thus, Orrell refers to how an unreflective search for unique aesthetic criteria can end in an appealing and simultaneously *formal and sterile order*.^70^ In various branches of science, but especially economics, this order format is portrayed, according to Orrell, as ‘beauty, clad in impressive looking mathematics’.^71^ Such processes lead to the following equation: *beauty = formal beauty*. If we choose to set aside these problematic aspects, we return to the original question: What is found ‘in the shadow’ of this beauty-seeking activity which manifests itself in an assumed formal and sterile order? In the previous section, we looked at how Bohm emphasised a deeper and implicate order. For Orrell, it is a matter of the following shift in focus:

To better understand the universe and our place in it, we are going to need more than telescopes and atom-smashers. At the heart of the transformation is a shift in aesthetics, from order and symmetry to something more *complex, organic, and messy.* The *structures to be erected* will be fluid and curved instead of square and static. Symmetry and perfection will be seen as special cases, rather than the authors of the universe. We will learn to celebrate qualities such as duality, mutability, and asymmetry—not just in physics or in science, but in our entire world view.^72^

Concurrent with Bohm’s mission, it is important for Orrell to underscore that our ‘gaze’ or perspective on the world is what is essential. This is ultimately the only thing that can allow for ‘structures to be erected’ and that concerns a *fluid, curved, asymmetrical, imperfect and incomplete reality*. That is, an approach based on a complexity aesthetics that in turn allows for a shadow landscape which, according to Orrell, can be understood and interpreted as ‘dark matter’. According to Orrell, this comprises not only ‘96% of the total mass-energy content of the universe’, but it can also be understood in different ways within various disciplines.^73^ Of course, this recognition of a new set of possible aesthetic preferences does not write off established scientific methods but indicates a next step in (knowledge) development.

It is important to note that neither does Orrell fail to acknowledge the beauty we can and must attribute to nature. However, this cannot be captured through a formal and sterile order, but it concerns something that may arise when we stop forcing all phenomena into a framework constituted of a mathematical model. When one allows for aesthetic ideals that concern asymmetry, this has to do with how Apollonian principles need to be balanced with Dionysian principles. Referring to Nietzsche, Orrell here demonstrates how art can be a corresponding point of reference for that which concerns the asymmetrical. If we enter into such thought experiments and allow a disruption of our world view, we will also have to tolerate *uncertainty*. We must open up to the unstable and fairly unpredictable, something that demands ‘more subjectivity and emotional engagement’.^74^ We need to open up to a ‘world of individual, changing, evolving, interconnected, implicit, incarnate, living beings within the context of the lived world’.^75^ This involves a shift from the mechanical paradigm to a recognition of that which is natural, organic and living, and where our traditional ideas of order are replaced in favour of a valuing of the whole over the parts, the context just as important as the abstractions and possibility just as important as predictability.

It is precisely such nuances related to the bodily aspect that are important when we follow Orrell’s reasoning in the direction of the medical domain. We can take our point of departure in the following: On the one hand, we have the natural psycho-physical order that is found in the well-functioning, healthy body: that is, order = health. On the other hand, we have the ‘order’ that medicine reveals in the unbalanced, dysfunctional body, and which the field itself labels *dis-order*. In other words, disorder = disease. That is, a disorder evaluated in relation to order. Paradoxically, we can regard this disorder as a state in which things are *not* in their place or in their proper place, but where the assumption is that things are in their ‘wrong place’ or imbalanced in certain predictable ways and *in accordance with* structures and patterns in established pathological explanatory models. Medical success depends precisely on the possibility that this *order of disorder* can be discovered, analysed and managed based on an evidence-based *bio-medical model* and carried forward by a present, but non-thematised *bio-mechanical aesthetics* – a calming and reassuring kind of order. We can unconditionally acknowledge that this order, for the most part, is decisive and lifesaving.

However, much of the medical community acknowledged long ago that the biological foundation and the bodily machine aesthetic, brought forth by new technology and ideals of *perfection*, cannot stand alone.^76^^,^^77^ It recognises, for example, that the biopsychosocial and patient-centred approaches are relevant when the field is tested in clinical practice. However, the biological framework is still strong, and what frightens many medical students I come across in my work is that they have already encountered many complex symptomatic profiles among patients and will continue to face many more. This may include mind-body and meaning-bearing forms of expression which even after examination cannot be captured using the exact (biological) knowledge students have acquired. They are thus confronted with what we may consider some kind of medically *dis-ordered disorder* which we can relate to Orrell’s *non-sterile, asymmetrical, unique, complex, organic and messy universe*. It thus is not difficult to understand that medical students and recently trained doctors may associate this human diversity and its complex expression with the “*unbeautiful”.*^78^

It is often in dialogue, in the actual clinical encounter, that these demanding expressions of the suffering person reveal themselves. Controlled by learned procedures for targeted and effective conversations, a communicative ‘machine aesthetic’ can easily become the preferred entrance and exit to this conversation. In many contexts, this approach yields satisfactory results. On other occasions, the clinician finds little support in the calming aesthetic of communication tools and instead perceives him/herself as unable to find a way out in the encounter with the person in pain. The familiar statement by patients ‘he/she doesn’t see me’ may be an expression of precisely such events.^79^ Incorporating ‘the living’ into such encounters requires not only time, but also an acceptance of asymmetry. Orrell elaborates on this as follows:

*Asymmetry is also a kind of marker for life... Just as symmetry is linked with unity and stasis, asymmetry is linked with plurality, change, and anything with a pulse.* Of course, one can always assume that any observed asymmetry corresponds to the accidental breaking of some other perfect symmetry, which is the standard procedure in theoretical physics, but the deeper question is about aesthetics: are we inventing symmetries where they don’t exist because we are convinced that they are beautiful according to conventional scientific standards?^80^

Orrell argues that symmetry can be linked to unity and stillness, which also means that which can be affirmed. Acting under time pressure, and with access to a comprehensive toolkit, healthcare professionals attempt to fulfil their mandate by applying their entire repertoire in order to reveal possible and fixed points for identification of illness – a possible symmetrical asymmetry (or ordered disorder). Sometimes, however, the problem is that the main clues cannot be traced to the physical and measurable heart rate linked to the circulatory system, but the ‘heart rate’ that may be incorporated and built into a rich story and a symptomatic plurality that express the living and the dynamic – and which precisely cannot easily be deciphered.

In conclusion, we might mention that Orrell can be criticised for casting a wide net, both in his treatment of the philosophy of science and in the range of disciplines that he covers, such as philosophy, mathematics, physics, sociology and economics. Another objection may be that in his criticism of our search for ‘the perfect model’ and our infatuation with numbers and mathematical equations, he fails to adequately highlight how projects underlying a mechanistic paradigm *also* pertain to an open, creative research process in the enigmatic, ‘non-sterile’ and ‘living’ fields of both physics and medicine, which frequently deal with outcomes that cannot be known. Likewise, we should not downplay how nature and even our bodies are not only organic, living and asymmetric, but also in *certain contexts* can and *should* be understood and treated as ‘mechanic’ – as analysable, divisible, predictable and reparable, or what in Svenaeus’ (2022) terms can be described as ‘good objectification’. However, such critical assertions do not alter Orrell’s main message that the ‘symmetrical” paradigm predominates, usually at the cost of a corresponding inclusion and respect for that which concerns the asymmetrical and “the living”’.

## Entering paradoxes - the order of knowledge and the order of charity

The starting point for the further examination here will be one of Jean-Luc Marion’s more recent contributions, the book *Negative Certainties *(2015). Like the previous contributors, Marion’s starting point is certain challenges related to our way of viewing, interpreting, and explaining the world. A key element here is thus *the object*, which he places in a larger context of knowledge. The emphasis is on how one can, in a scientific context, as well as in other and more practice-adjacent contexts, primarily explore, focus on, and intervene in the aspects in one’s field or ‘scope’ which can be reduced to the criteria of scientific models or manuals and tools. According to Marion, this means ‘*the field of that which can be put in order (the order that orders according to knowledge, indifferent to the natural disposition of the presumed essence of the entities), that is to say, all that which can be modelled: and therefore the field of what can be measured*’.^81^ The complete set of criteria requires that the ‘field’ in which one operates is transformed into a manageable *object format* which is adapted to the rationale of the exploring instrumentarium.

In this context, the object refers to something which is characterised by measurability, predictability, and reproducibility, and which is external to the thinking mind or subject. This is something we face (see the German *Gegenstand*), which is often clarified by and for the targeted, active, creative and goal-oriented person. Marion finds that this aspect (see the previous passages regarding Bohm and Orrell) leads to the following summary: ‘It follows that the object is never defined in itself nor by itself, but always by the thought that knows it in *constructing* it’.^82^ It is precisely this subject’s ‘grasp the world capacity’ that Marion wants to challenge by incorporating the opposite of the object: *the phenomenon,*in other words, what in principle can be ascribed to characteristics such as subjective, unreliable, imprecise, unmanageable, and unstable. Consequently, according to Marion, there is a *knowledge-based asymmetry* in the relationship between object and phenomenon, where the object holds a hegemonic position in relation to the phenomenon. This is elaborated on as follows: ‘According to its mode of knowledge, its ratio cognoscendi, the phenomenon of the object type prevails indisputably over the non-objective phenomenon, which it devalues as uncertain, imprecise, and confused – in short, as being at the margins of knowledge and quasi-irrational: subjective.’^83^

The phenomenon is thus located at the edge of what simply could be described as reliable knowledge. However, it is worth noting that what Marion refers to here is the object as ‘*the phenomenon of the object type*’ and the phenomenon as ‘*the non-objective phenomenon*’. Here Marion insists that in reality we are dealing with a univocal phenomenality. Here the phenomenal, unlike what is usually expected, must be considered a unifying and common starting point. The phenomenon’s status consequently can no longer be written off as being a ‘failed object’, but instead be highlighted as *what always exists, rich and full of abundance from its given starting-point.* This means that the non-objective phenomenon, compared with the phenomenon of the object type, ‘arises from itself, without any warning to prepare us, and without any repetition to accustom us to it, it imposes itself as an actuality without cause, autonomous, spontaneous, fully realised of itself and always in advance of any knowledge we might later glean from it’.^84^ Based on such characteristics, Marion chooses to call these *saturated phenomena*. This means phenomena that are rich, full of abundance, and where intuition ‘floods’ or exceeds the intentionality. These are phenomena that cannot be attacked, mastered, or handled, but which are abundance phenomena that more clearly ‘grab us’. Marion describes several conspicuous examples of such as follows:

Now, such phenomena [saturated with intuition] even if their phenomenality is characterised by an exceptional excess (the intuition exceeds the limits of the concept), are nothing if not banal and frequent in our experience… First, *the event* which happens in the superabundant intuition of history... Second, the *idol* or the excess of sensible quality, which overflows what the organs of perception can receive and handle… Third, *the flesh*, or more precisely my flesh, insofar as in it I undergo the experience of feeling itself…Finally, the *face of the other* (or the icon) as it escapes from the inert visibility of a subsistent object in the world...’ and instead ‘speaks to me, since it provokes and convokes me through a reversed intentionality, which henceforth goes from it toward me’.^85^

However, Marion points out that such phenomena cannot be considered selected special cases or exceptions. Phenomena being saturated (intuition overrides intention) must instead be considered the general situation, in other words, what springs out of the paradigm of givenness. In this, we are not primarily producers of meaning but recipients, the gifted, and our challenge is ‘recognizing the meaning that the phenomenon itself gives from itself and to itself’.^86^ What then is the place of saturated phenomena in terms of order? In an attempt to clarify matters, I will highlight what may be the main nexus in our lives: love. In his book *The Erotic Phenomenon *(2007), Marion points out that this phenomenon has been overlooked or neglected, in philosophy.^87^ Part of the reason for this may be the inability to capture this phenomenon in the format of an object, submit it to the mechanisms of causality, enter into a transaction (following the model of economics), or to measure or predict it. Love not only requires being released from such guidelines and ties, it also evades a specific rationality – ‘one that fits with the things of the world, *objects of order and measure*, and with their calculation and their production’.^88^ Instead love falls under a different form of rationality, a ‘greater rationality’ – ‘that which does not limit itself to the world of things nor to the production of objects, but which instead rules our hearts, our individuality, our life and our death, in short that which defines us deep down in all that concerns us in the final instance’.^89^ Love *‘ruling’* our lives implies what Marion (1999) refers to in a few rare instances as the *order of charity*.^90^ With reference to Pascal, he particularly emphasises the following aspect: ‘To see the ‘″order of charity’’ one has not so much to know a new object, as to know according to a new condition, loving’.^91^ This means that love carries its own ‘supreme order’ which ‘remains invisible to the flesh and to the spirit, to powers and to science’.^92^ The ambition to capture love in scientific explanatory models may consequently be suspended, and instead we may allow this to be a given and abundance-like life event we may be charged with. As Peters and Peters (2013) add in timely fashion, love is thus ‘a fickle thing, fickle because it is not a thing’.^93^

If we extend Marion’s thinking more concretely into the field of medicine, the starting point may be the distinction between the object (the object phenomenon), which is the colonising phenomenon which dominates the stage, and other phenomena (saturated phenomena), which are placed in the background of that which refers to a strengthened asymmetry. In many *health* contexts, this asymmetry may be considered necessary, effective, and adequate depending on the situation. A doctor’s primary mission is undeniably to detect whether there objectively is a serious illness and to implement the adequate intervention. This practice requires the use of scientific models, seeks support from best evidence, applies manuals, and on the whole applies this to a *field* (the human body) which precisely *can be ordered*. A context in which this is particularly meaningful is the A&E departement of a hospital. The focus now is on bodily functions, visible harm, and the condition of individual organs, in other words a process characterised by *necessary fragmentation and reduction of the body to a manageable object format.*^94^ This means that the body here must be transformed ‘to that in it which can be known with certainty’.

At the same time, we must affirm that as long as the one in need is a person, in Marion’s words the gifted, whose body is touched through the experience of him/herself as flesh, and existing in a flow of events, the object approach and the affirmation of the object will represent a *decimated and reduced version of this basic wealth of phenomena.* This means that what one finds are *object-funded derivations of expressions of life that carry and give meaning in a person who is in pain or suffering.*^95^ These rich expressions of life will *always* be in play *before* a healthcare professional enters into the arena and transforms it into a manageable format. When the situation is no longer acute, requiring a resolute object approach, the helper mentioned above may find that the pain cannot be measured and there is no obvious form of intervention. We therefore face an object of study that cannot be captured in the format of an object. The challenge for the medical professional is therefore to be able to distinguish between when the medical reduction and the objectification are required or critical to life, and when in other contexts they may be obstructive tools. This means distinguishing between what is necessary and adequate reduction and a need for attention that takes into account that to ‘classify a man is to downgrade him *as human*, because he could not be classified any other way than according to an order and a measure (models and parameters) that come to him from elsewhere – namely, from the workings of my rationality’.^96^

In relation to the clinical encounter, this is precisely an ‘arena’ in which the above-mentioned considerations, dilemmas, and paradoxes may manifest themselves. In the phenomenological tradition, of which Marion is also undeniably a part, it has been emphasised how such encounters imply unavoidably that *the subject’s life-world* is ‘in play’ (Husserl 1970), understanding is made possible through a *fusion of horizons* (Gadamer 2013) and that we encounter the *face* of the other (Levinas 2003), including ethical demands (Løgstrup 2020) which also allow for an *infinite hermeneutics* (Marion 2002b), and which nonetheless involve the meaning of *bodily* presence (Merleau-Ponty 2003). These contributions in the history of philosophy remind us of how instrumental attempts to grasp and control human encounters, including dialogue, can be challenged or ‘put under pressure’. Many of us have also experienced how conversations can take a turn that digresses from the desired and established course and throw us defenceless, temporarily or permanently, into an unpredictable upheaval that reveals the thoughts’ shortcomings and the experience of being under emotions’ power. This reminds us that dialogue can unfold in ways that are not a product of our planned efforts, and that conversation, as an event, can ‘establish’ or ‘fix’ an order that gives in, or orders ‘for itself’, in ways that violate any idea we may have had about a process in which *the things will find or be assigned their rightful place*. In this, we may also find that our usual coping strategies come up short and that attempts to regain control and take the wheel do not bear fruit.

Drawing on Marion’s Levinas-inspired approach, the question of *the Other* and the face of the other again gains relevance. A prerequisite for a conversation that precisely does not anticipate and predict the person in need instead allows the healthcare professional to be summoned – ‘in silence’. In Levinas’ original words, this is a matter of ‘*The order that orders me.*.’.^97^ If one is attentive to this summons, one may find that the conversation’s centre of gravity shifts from the helper’s analytically exploratory gaze to the other himself. This is a matter of an appeal, what Marion expresses as ‘This face, like this gaze, gives me nothing to see—but gives itself by weighing on me’. He elaborates: ‘The gaze that the Other casts and makes weigh on me therefore does not give itself to my gaze, nor even to be seen—this invisible gaze gives itself only to be endured’.^98^ The healthcare professional changes here from being a distanced watcher, to a *witness*, a witness to ‘something’ revealing itself: something which is not decided in advance, constituted or produced by the giver of help. In this summons and in a glimpse of varying durations, which can appear to be un-understandable and ungraspable, ‘I learn of myself from what the gaze of the Other says to me in silence’.^99^ Marion concludes by reminding us that ‘the concept of witness finds its full phenomenological legitimacy only when related to the saturated phenomenon of the Other, who alone can constitute me as his own because he *precedes me in the order of manifestation*.’^100^

## Conclusions

Given the interpretation above, we have a final and obvious opportunity here, namely to collect different statements, positions and concepts into an expression that communicates what we can choose to understand as the quintessence of order: *the table*.

**Table 1:** Summarizing order

	Kuntz	Bohm	Orrell	Marion
Subdiscipline	Epistemology (ontology)	Ontology (epistemology)	Aesthetics (epistemology)	Phenomenology (ethics)
Qualities of order	Clarity	Simplicity	Symmetry, beauty	Measurability
Problems of order	Complexity	Fragmentation	Sterility	Objectification
Key concepts	Formats of order Order of order	Explicate and Implicate order	Formal order Organic order	Order of knowledge Order of charity
Medical challenges and dilemmas	Taxonomies and dualities of order	Psyche and soma	The order of disorder	The object-body and the other as a saturated phenomenon

In various ways, the presentation in a tabular format ensures that things fall into their place or into ‘their proper place’. It clarifies their mutual relationships, and we see *the contours* of how we can gain both an overview and a better grasp of the matter of order. In a positive and perceptible sense, the table highlights an aesthetic in which a calming elegance could be a point of reference. It separates, differentiates, categorises and helps make the topic explicit and available in all its complexity and manyness. It very efficiently summarises some of the key points that have been described above, and in Heidegger’s words, it presents ‘a plethora of information’ that appear to respond to the task at hand.^101^ The challenge however, as Heidegger also reminds us in a rare discussion of this topic, is that although we imagine that we are responding to *and creating order*, we can in fact be led astray.^102^ He elaborates:

The syncretistic comparison and classification of everything does not of itself give us genuine essential knowledge. Subjecting the manifold to tabulation does not guarantee a real understanding of what has been ordered. *The genuine principle of order has its own content which is never found by ordering, but is rather already presupposed in ordering*.^103^,^104^

This table must hence be understood as something more than, and different from, an ordinary table, i.e. as something more than a mere presentation of facts and data in a system of coordinates. Hence, it does not document that we have concluded our thinking about order; it is rather an expression that enables us to *start thinking about and thinking into what order should be all about*. We are here provided with a set of formal indications, i.e. some linguistic clues (signposts) that set a direction. About the use of these, Heidegger (2004) says the following: ‘In the formal indication one stays away from any classification; everything is precisely kept open. The formal indication has meaning only in relation to the phenomenological explication’.^105^ The explication that here is referred to quite specifically is *order as a saturated phenomenon*. This means that the key concepts in the table point towards a saturated universe of meaning, where order simultaneously can be conceived of as *a pivotal point for* homo ordinans, *a multidisciplinary concern, a problem-generating activity, a quality, a concept, an object amenable to scientific inquiry, an exploratory point of departure, something we can point towards, or most importantly, a phenomenon that in itself points (towards other meaningful phenomena)*. Furthermore, and far more crucially, these key concepts point towards some normally hidden fundamental preconditions for our observation and investigation of order. These remind us that the ‘matter’ of order is also about something more than order itself and invite us to ponder the deep structures of the universe and the world that challenge order (Bohm), how we are embedded in an organic, asymmetrical whole (Orrell), and how we incessantly find ourselves to be recipients of phenomena that embody a surplus that may exceed and overwhelm our ability to respond analytically (Marion).

If we instead choose to consider the table as representing a final framing and determination of order, we risk that our ordering project will be *loveless: loveless because it is ordered without concern for that which is ordered.* Heidegger offers a crass description of this lovelessness in his later work, *The Event*:

Human beings themselves, like the organised superman, seem to dominate everything and are dispropriated of the last possibility of their essence: they can never recognise in the extreme blindness that the human forgetfulness of being, a forgetfulness brought to maturity along with the abandonment of beings by being, leaves human beings without a sense of plight insofar as it compels them to think that the *ordering of beings* and the *instituting of order* would bring about the substantive fullness of beings, whereas indeed what is assured everywhere is only the endlessly self-expanding emptiness of devastation.^106^

Heidegger argues here that in blind faith to our ordering efforts, we believe we assert, manifest and complete the being in all its fullness, while in reality we leave the same in a well-ordered and resoundingly silent emptiness or vacuum of meaning. If we heedlessly and in an unreflective manner apply our order formats to phenomena that are not intended for such ordering in our search for knowledge, this *restriction of meaning* can become the end result. Since the motivation for writing this paper was to increase our understanding of order, the table is hence not designed to capture, but rather to enable an *expansion of meaning*.

As regards medicine, we have seen that this restriction of meaning is appropriate and legitimate, especially when people suffer for reasons that only the discipline’s instrumentarium can address. Finally, we may thus opportunely call attention to an ongoing pandemic that started with many unknown factors, complexity and uncertainty, and that thanks to the well-developed systematism of modern medical science has brought clarity to virus types, infection routes and protective measures, and devised an effective vaccine. In this context, the expression ‘getting things in order’ becomes especially meaningful. Similarly, including in regular clinical practice, we need to stick to the view that searching for what can be subsumed under a relevant diagnostic label, a well-tested surgical procedure, a quality-assured guideline or a reliable testing regime constitutes crucial clinical evidence. This is what the medical practitioner is trained to do, must do and ought to do. When the clinician receives the results from a laboratory’s broad-spectrum analyses, frequently presented in a tabular format, vitally important parameters may become clear. This ability to analyse, differentiate, interpret and categorise, to reduce complexity and activate doctrinaire knowledge, can accordingly be regarded as a *positive capability*. It is crucial in the efforts to reestablish bodily balance or harmony in a suffering human burdened by disease. To the extent that a disciplinary affinity for order and what can be ordered is involved, it here delivers at its best a meaningful and manifest *positive certainty*.

Becoming *aware* of the discipline’s ordering instrumentarium, the ordering activity and its consequences constitute a major challenge for the discipline. In the scientific realm, this is not a matter of losing faith in the method, but in discovering whether one blindly applies scientific methods, regardless of whether these methods are suited to the object of study. It is not a matter of a general distrust of empiricism or empirical data, but more an unreflective insistence on empirical control of practice (evidence based practice). For the more practically oriented clinician, this awareness-raising process is similarly important. In the encounter with suffering patients who do not suffer in obviously medical or known pathological ways, an unthinking affinity for systems that produce order may impede or displace the meaningful life expression that the patient brings to the table. To become aware of and reflect on this, even medical students need to be reminded that there are alternatives to hopelessness and paralysis. What is sometimes demanded is for the medical practitioner to some extent to make themselves “available for” a *negative capability*. This means ‘to be open to the actual vastness and complexity of experience, and one cannot possess this openness unless one can abandon the comfortable enclosure of *doctrinarian* knowledge”.^107^ If we permit such aspects of our world of experience to manifest themselves on a comprehensive scale, we do not gather knowledge about, but are shaped and characterised by, a particular form of *negative certainty*. This is not dubious or mysterious, nor can it be understood as an epistemic vacuum. It is based on us re-transcribing and transforming the phenomena that are previously regarded as objectivising ‘into primordially given phenomena, because they are giving themselves in themselves’.^108^ Of course, this negative certainty concerns not only the field of medicine. It can be meaningful in other situations, such as our attempts to address the state of the planet and the ongoing environmental and climate crisis.

As a final shift, we may therefore choose to view the complex and composite nature of order as a standing invitation, one that invites us to disrupt established perceptions to the extent that we also risk viewing ourselves and the world in a different light. The possibilities are many, as Chmielewski (2020) reminds us, because ‘the need to discover, establish, impose, perfect and abolish order, never expires in man’.^109^ Nevertheless, we must live with the fact that order is demanding to capture. The conceptual difficulties are linked to the omnipresence of order, and Bohm (1980) reminds us that: ‘in its totality [it] is evidently ultimately undefinable, in the sense that it pervades everything that we are and do (language, thought, feeling, sensation, physical action, the arts, practical activity etc.).^110^ In the same vein, and referring to one of Heidegger’s later contributions (1977), he reminds us that ‘the essence of technology is by no means anything technological’. Correspondingly, and by way of conclusion we can suggest the following: the essence of order is by no means anything orderly or ordered. 111

## Footnotes

1 Rotenstreich (1972, p. 121).

2 ‘Order, n.’ *OED Online*. Oxford University Press, December 2019. Web. 14 January 2020.

3 Chmielewski (2020, p. 3).

4 Kuhn (1968 p. 446).

5 Aristotle (2018) p. 141.

6 Lang (2007) p. 3.

7 Bayer (1998) p. 127.

8 Ibid. p. 128.

9 Pascal (2011) p. xvii.

10 Merleau-Ponty (1963) p. 133)

11 Berrill (1966) p. 515.

12 Orrell (2007) adds, ‘The need to search for order and pattern seems to be a fundamental characteristic of human beings. Science, like religion, is a way of structuring and making sense of the world, a kind of bastion against chaos’ (p. 67).

13 Chmielewski (2020) p. 3.

14 *OED Online*. Oxford University Press, March 2020. Web. 25 April 2020.

15 Gabriel (1999) touches upon this in a description of how ‘regulations, routines, time-tables, accounts, records, regularity and predictability’ concern a predetermined apparatus that ensure order. In this bureaucratic system we find that ‘creativity, emotion and spontaneous interaction are profoundly threatening phenomena, generating acute anxiety…» (p. 65).

16 In connection with this, Rothenstreich (1972) notes that ‘philosophical knowledge is a deliberate knowledge, it is plain that philosophical knowledge is concerned with *knowledge arranged in order*. The philosophical question concerns the possibility of arranging knowledge in order and this is known as systematic order or a system’ (p. 133).

17 This contrasts sharply with traditional philosophy and the age-old ideals in which the task of philosophy is there for these real-life challenges and to be all-encompassing, deeply explorative and ‘thick’. (Solomon 1999, p. vi).

18 See, for example Garvey (2008), Madu and Kuei (2012).

19 Svenaeus (2022) p. 1.

20 Ibid.

21 On this point, Marcel (1973) reminds us that ‘there is an order where the subject finds himself in the presence of something entirely beyond his grasp. I would add that if the word “transcendent” has any meaning it is here—it designates the absolute, unbridgeable chasm yawning between the subject and being, insofar as being evades every attempt to pin it down’.

22 Kuntz (ed) et al. (1968)

23 Ibid. p.x.

24 Ibid. p.xxxix.

25 Ibid. p. xxvi.

26 Ibid. p. xxvii.

27 Ibid. p. xxviii.

28 Ibid. p. xxix.

29 Ibid. p. xxxiii.

30 Ibid. p. 452.

31 In his study, Kennedy-Martin (2015) expresses this as follows: ‘In the majority of studies included in this literature review it was concluded that patient samples in cardiology, mental health, and oncology RCTs are not broadly representative of patients encountered in everyday practice’ (p. 11).

32 See for example Milgrom (2021.)

33 See for example Svenaeus (2013), Beresford (2010), Cassels (2012), Eriksen (2012), Eriksen (2013), Eriksen (2019).

34 Franco et al. (2020)

35 Brindley et al. (2014) provides a useful overview of the most important of these (the GREAT technique, the LAURS technique, he VALUE framework and the SPIKES technique).

36 Lorand (2000) p. 21–22.

37 Rotenstreich (1972) p. 196.

38 Chmielewski (2020) accordingly argues that ‘Norms of the cognitive regime [intellectual and cognitive order] thus fulfil a disciplinary task; they allow certain ways of seeing or speaking, while prohibiting other ones.’ (p. 7).

39 The term *radical* suggests that Bohm’s theories have been the subject of controversy both in philosophy of science circles and in quantum physics. Nevertheless, and as the physicist Talbot (2020) states; ‘50 years on, questions raised about the direction and philosophical assumptions of theoretical physics show that Bohm’s arguments still have contemporary relevance’. In recent decades, Bohm’s theories have primarily been further developed by the Finnish philosopher of mind Pylkkänen (1989, 2001, 2007, 2010), the quantum physicists Hiley & Peat (1987), Bohm and Hiley (1993) and the physicist Freire (2019). It is also worth mentioning that Bohm’s crossing of disciplinary boundaries has also inspired the works of theologians such as Peters (2016) and Keller (2015), sociologists such as Tamdgidi (2020) and Scheff (1990) and mathematicians such as Dürr and Teufel (2009).

40 See Bohm and Hiley (1993).

41 Nichol (ed.) (2005, p. 3).

42 The interview is from the documentary ‘*Art Meets Science and Spirituality in a Changing Economy – From Fragmentation to Wholeness’*. Topics from the symposium, Art Meets Science and Spirituality in a Changing Economy, Amsterdam, 1990. Web: https://www.youtube.com/watch?v=hfHzfonAgX4.

43 Ibid.

44 Bohm (1980, p. 3).

45 Ibid. p. x.

46 Ibid. p. xviii.

47 Bohm (1996) p. 7.

48 Bohm (1980), p. 262.

49 Croicu et al. (2014).

50 Kroenke et al. (2010).

51 Bohm (1980), p. 265.

52 Later, and in the article ‘*A new theory of the relationship of mind and matter’*, Bohm (1990) expands on this. The most crucial point is that both mind-like qualities of materiality and the material qualities of mind are a part of the aforementioned unbroken whole. In this whole (the implicate), ‘mind’ and ‘matter’ are intertwined via what Bohm calls *interaction and participation: ‘*For the human being, all of this implies a thoroughgoing *wholeness*, in which mental and physical sides *participate* very closely in each other. Likewise, intellect, emotion, and the whole state of the body are in a similar *flux* of fundamental *participation*. Thus, there is no real division between mind and matter, psyche and soma. The common term psychosomatic is in this way seen to be misleading, as it suggests the Cartesian notion of two distinct substances in some kind of interaction’ (p. 284).

53 Lissoni (2021), p.9.

54 See also Pescatori et al. (2015), who highlight the way in which ‘the psycho-neuroendocrine-immune approach relies on the concept of considering diseases from a *holistic* point of view’ (p. 269).

55 See e.g. Vogt et al. (2016) and Ziebarth (2016).

56 Woods (2015).

57 On this topic, Bohm (1951) remarks that expectations about intelligibility, measurability and conceptual simplicity admittedly correspond well with an established mechanistic worldview, but that such requirements disregard that ‘the undivided universe’ cannot be captured at a classical level of accuracy and description. (p. 167).

58 Seager (2018) p. 1.

59 Ibid.

60 This topic is also addressed by others such as Weindberg (1994), Lindley (1994), and Stewart (2007).

61 Among different sources, Orrell here refers to Aquinas and Joyce: ‘Aquinas proposed three attributes that make an object beautiful: *integritas, consonantia, claritas* – or, as James Joyce translated them wholeness, harmony, and radiance’ (Orrell 2012) p. 26.

62 Referring to philosopher Mark C. Taylor, Orrell explains that machine aesthetic concerns ‘the idea of the grid. Grids are straight, static, simple, symmetric, uniform, and functional. They only have a single scale because each square of the grid is the same size’. Ibid. p. 244.

63 Ibid. p. 39 (my emphasis).

64 Ibid. pp. 236–237.

65 Ibid. p. 287.

66 Ibid. p. 207.

67 Ibid. p. 253.

68 Ibid. p. 288.

69 Ibid.

70 Orrell is referring here to R.D. Laing: ‘Galileo’s program offers us a dead world: (…) out go sight, sound, taste, touch, and smell, and along with them have since gone esthetic and ethical sensibility, I values, quality, soul, consciousness, spirit’ (p. 242).

71 Ibid. p. 235.

72 The phrase ‘the structures to be erected’ does not suggest that Orrell is disregarding each and every form of structure or order format, but emphasises that this does not satisfy the demands we usually make. Ibid. p. 275.

73 Ibid. p. 202.

74 Ibid. p. 215.

75 Ibid. p. 267.

76 In relation to perfection and health, see e.g. Callahan 1998; Brustein 2014; Sirois et al. 2016.

77 The search for perfection may manifest itself in several ways. That which is in relation to ‘formal beauty’, where symmetry, unity, harmony, and the straight are in focus, may promote (a) ‘perfect models’ of wellness and healthiness being unrealistic goals for seeking souls (see the normative figures for body mass index – BMI – set by the authorities); (b) ideals of perfect beauty resulting in excessive consumption of cosmetic surgery; (c) ideas of ‘perfect health’, understood as an absence of pain, leading to a constantly expanding menu of diagnoses becoming a nexus of self-identification; and (d) technological solutions giving us ways to self-monitor health parameters (‘do-it-yourself health care’), which may reinforce an imbalanced preoccupation with one’s own health.

78 Shattock et al. (2013) state that ‘Current medical training fails to equip doctors to engage with MUS [medically unexplained symptoms] and potentially fosters the development of *unhelpful views* of these patients’ (p. 249). Howman et al. (2016) add the following: ‘Managing MUS is a common problem for GP trainees and results in a disproportionate amount of anxiety, frustration and uncertainty’ (p. 1).

79 It must be added that many doctors criticise the authorities’ guidelines and the frameworks of healthcare institutions, which precisely require *clinical* communication in the true sense of the word (here: efficient, cool and unsentimental).

80 Orrell (2012, p. 191)

81 Marion (2015, p 2).

82 Ibid. p. 23. (emphasis added).

83 Ibid. p. 159.

84 Ibid.

85 Ibid. pp. 203–204.

86 Marion (2002a).

87 According to Marion, the very maxim of philosophy, the love of wisdom, has gradually been reduced to the love of knowledge or even the hoarding of knowledge.

88 Marion (2015), p. 5 (emphasis added).

89 Ibid.

90 Marion’s (1999) reference here to the ‘order of charity’ is, as in other contributions, a direct reference to Pascal’s third order (the order of charity).

91 Ibid p. 316.

92 Marion (2019) p. 46.

93 Peters and Peters (2013) p. 1.

94 This often involves mapping vital life functions, such as heart rate, temperature and blood pressure.

95 This explanation further corresponds to Bohm’s theory on explicate and implicate order: ‘So we are suggesting that it is the implicate order that is autonomously active while, as indicated earlier, the explicate order flows out of a law of the implicate order, so that it is *secondary, derivative, and appropriate only in certain limited contexts.* Bohm (1980, p. 235).

96 Marion 2015, p. 26.

97 Levinas (1991).

98 Marion 2002a.

99 Ibid.

100 Ibid.

101 Heidegger (2010) p. 50.

102 Heidegger (2010) touches upon this topic in connection with the need for development of ‘a natural concept of world’. Among other things, he makes reference to how the ontic sciences, i.e. psychology, ethnology and sociology, have been unable to meet this challenge (p. 52).

103 Ibid. p. 48 (my emphasis).

104 In connection with this quote from Heidegger, Thompson (2005) provides an important specification: ‘In other words, that which establishes a system (or defines a concept) cannot be grounded by the system it establishes (or the concept it defines), no more than an axiom can be proved by the theorems derived from it’ (p. 52).

105 Heidegger (2004) p. 44.

106 Heidegger 2013, p. 141.

107 Ou (2009), p.2.

108 Marion (2015) p. 202.

109 Chmielewski (2020) p.5)

110 Bohm (1980) p. xvii.

111 Heidegger (1977) p. 4).
